# 2,3:4,5-Di-*O*-iso­propyl­idene-β-l-fructo­pyran­ose

**DOI:** 10.1107/S2414314626011247

**Published:** 2026-10-30

**Authors:** Tomoko Takashita, Kei Takeshita, Akari Otabara, Tomohiko Ishii

**Affiliations:** a Graduate School of Science for Creative Emergence Kagawa University, 2217-20 Hayashi-cho Takamatsu Kagawa 761-0396 Japan; b FUSHIMI Pharmaceutical Co., Ltd., 307 Minatomachi, Marugame, Kagawa 763-8605, Japan; Katholieke Universiteit Leuven, Belgium

**Keywords:** crystal structure, rare sugar, l-fructose, acetonide, hydrogen bonding

## Abstract

The title compound, C_12_H_20_O_6_, crystallizes in the tetra­gonal space group *P*4, with two crystallographically independent mol­ecules in the asymmetric unit. Each independent mol­ecule contains one hy­droxy group that acts as both a hydrogen-bond donor and an acceptor. In the crystal, separate O—H⋯O hydrogen-bonding inter­actions connect four symmetry-related mol­ecules into cyclic tetra­meric assemblies around the crystallographic fourfold axes. The corresponding d-enanti­omer has previously been determined at room temperature [CSD refcode FOYTAO; Lis & Weichsel (1987 ▸). *Acta Cryst.* C**43**, 1954–1956] and redetermined at 173 K [Yathirajan *et al.* (2004 ▸). *CSD Communication* (FOYTAO01). CCDC, Cambridge, England]. The close agreement in space group, asymmetric-unit contents and crystal packing indicates that the present l-enanti­omer is isostructural with the previously reported d-enanti­omer.

## Abstract 2

The crystal structure of 2,3:4,5-di-*O*-iso­propyl­idene-β-l-fructo­pyran­ose is reported and compared with two previous determinations of the corresponding d-enanti­omer. In the crystal, the two independent mol­ecules are linked separately by O—H⋯O hydrogen bonds, forming cyclic tetra­meric assemblies.

## Graphical abstract

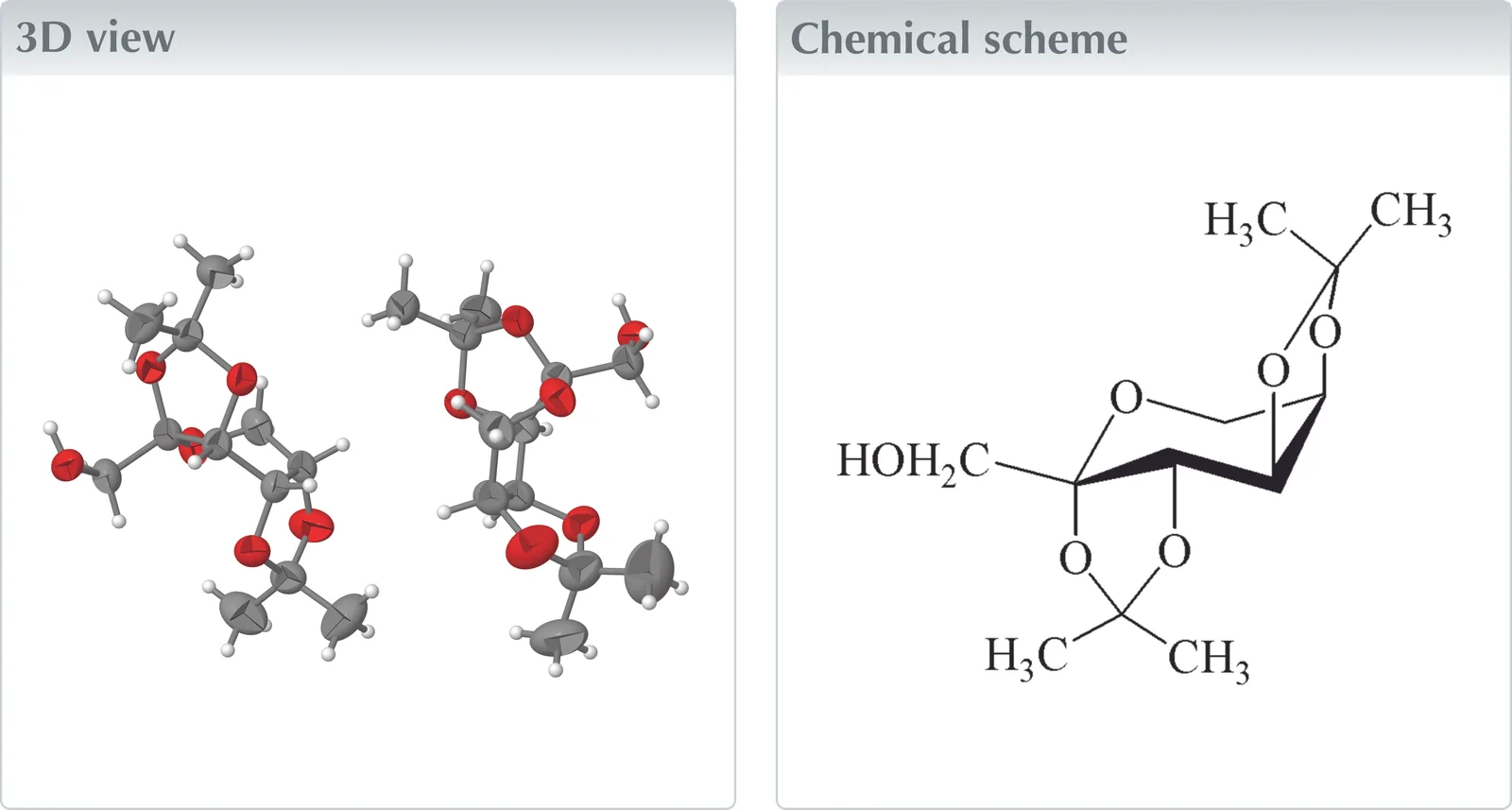

## Structure description

**Figure 1:**
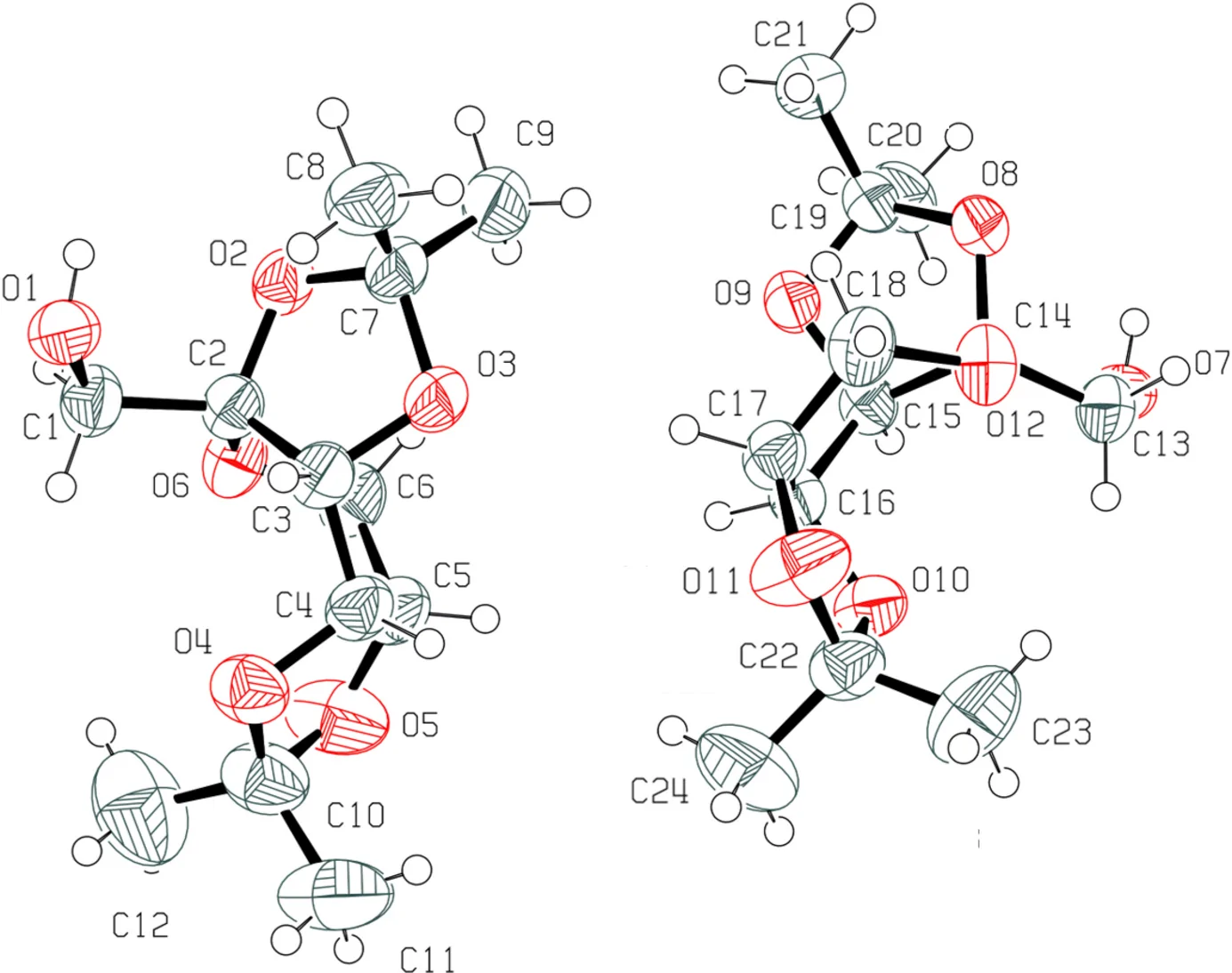
The two crystallographically independent mol­ecules *A* (left) and *B* (right) in the asymmetric unit of 2,3:4,5-di-*O*-iso­propyl­idene-β-l-fructo­pyran­ose, showing the atom-labelling scheme. Displacement ellipsoids are drawn at the 50% probability level, and H atoms are shown as small spheres of arbitrary radii.

The title compound, C_12_H_20_O_6_, was prepared by acetonide protection of l-fructose. The mol­ecular structure is shown in Fig. 1 ▸. The compound crystallizes in the tetra­gonal space group *P*4, with two crystallographically independent mol­ecules, *A* (containing O1) and *B* (containing O7), in the asymmetric unit (*Z*′ = 2). The two independent mol­ecules have closely similar mol­ecular conformations (r.m.s. deviation = 0.111 Å).

The crystal structure of the corresponding d-enanti­omer, 2,3:4,5-di-*O*-iso­propyl­idene-β-d-fructo­pyran­ose, was first reported at room temperature (CSD refcode FOYTAO; Lis & Weichsel, 1987 ▸) and was subsequently redetermined at 173 K (FOYTAO01; Yathirajan *et al.*, 2004 ▸). The structures were identified in the Cambridge Structural Database (CSD; Groom *et al.*, 2016 ▸). Both reported d-enanti­omer structures crystallize in space group *P*4, with two crystallographically independent mol­ecules in the asymmetric unit, as observed for the present l-enanti­omer.

The unit-cell parameters of the present l-enanti­omer at 296 K are: *a* = *b* = 21.5539 (14), *c* = 5.9556 (2) Å and *V* = 2766.8 (4) Å^3^. These values are close to those reported for FOYTAO at 295 K: *a* = *b* = 21.510 (10), *c* = 5.940 (3) Å and *V* = 2748.3 Å^3^. The unit-cell parameters reported for the low-temperature redetermination FOYTAO01 are: *a* = *b* = 21.4209 (15), *c* = 5.8879 (6) Å and *V* = 2701.7 (4) Å^3^. Its smaller unit-cell dimensions and volume are consistent with the lower measurement temperature of 173 K. The agreement in the space group, number of independent mol­ecules and overall crystal-packing arrangement indicates that the present l-enanti­omer is isostructural with both reported structures of the d-enanti­omer.

**Figure 2:**
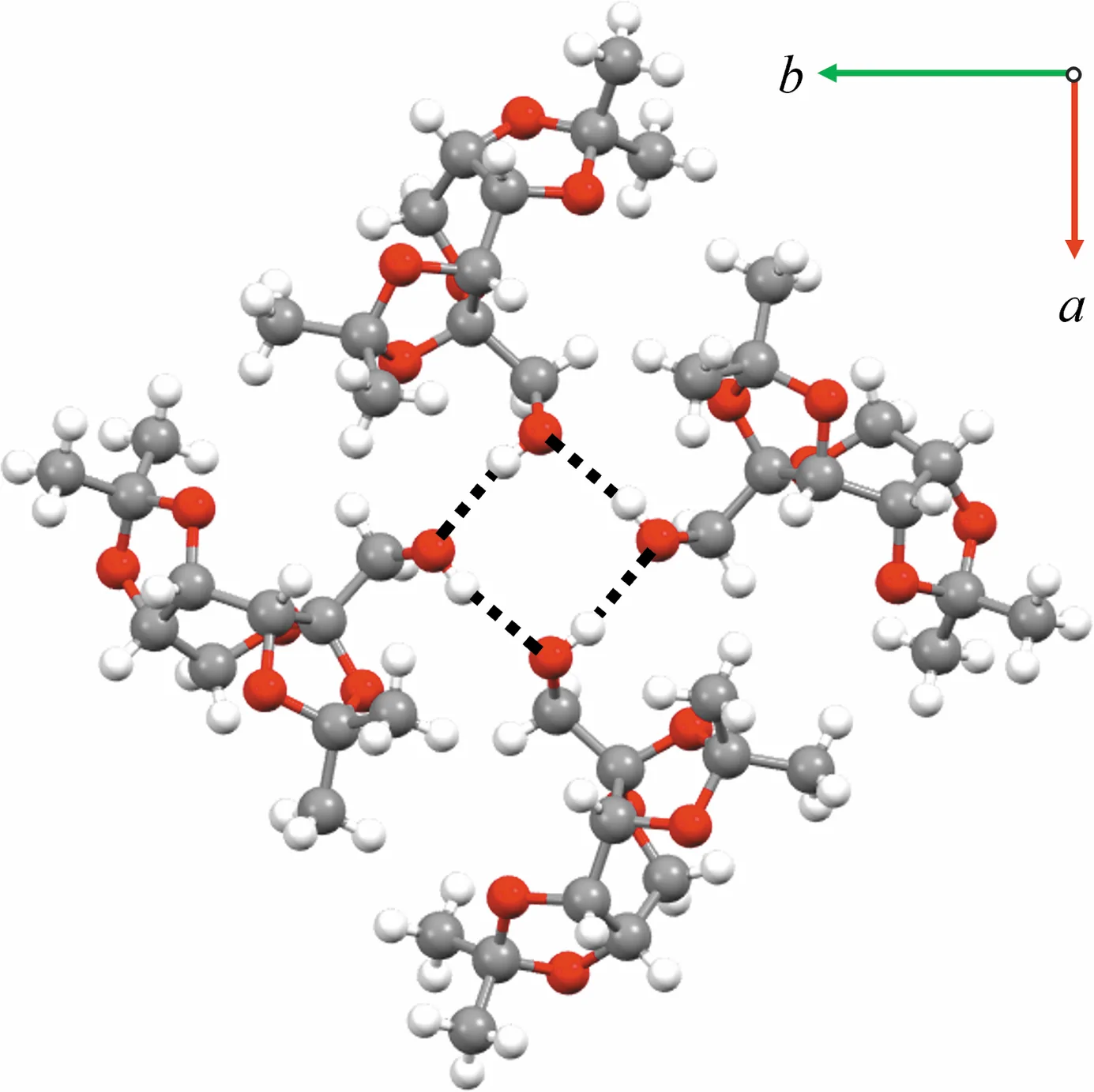
Cyclic tetra­meric assembly formed by four symmetry-related mol­ecules of type *A*, viewed along the *c* axis. O—H⋯O hydrogen bonds are shown as black dashed lines. Mol­ecules of type *B* form a separate cyclic tetra­meric assembly of the same kind.

In the crystal of the title compound, the hy­droxy group of each independent mol­ecule acts as both a hydrogen-bond donor and an acceptor. Mol­ecules of type *A* are linked by O1—H1⋯O1 hydrogen bonds, with an O⋯O distance of 2.776 (2) Å and an O—H⋯O angle of 166°. Independently, mol­ecules of type *B* are linked by O7—H7⋯O7 hydrogen bonds, with an O⋯O distance of 2.738 (2) Å and an O—H⋯O angle of 164°. Each hydrogen-bonding inter­action connects four symmetry-related mol­ecules around a crystallographic fourfold axis, producing two crystallographically independent cyclic tetra­meric assemblies (Fig. 2 ▸, Table 1 ▸).

## Synthesis and crystallization

A mixture of acetone, l-fructose and 2,2-di­meth­oxy­propane was cooled to 10°C with stirring. A 70% aqueous solution of perchloric acid was added dropwise, and the reaction mixture was stirred for 18 h. The mixture was subsequently neutralized with 1 *M* aqueous sodium hydroxide solution and adjusted to pH 7.

The product was extracted with ethyl acetate and water. Separation and recovery of the organic phase were repeated three times. The combined organic layers were dried over anhydrous sodium sulfate and filtered. The filtrate was concentrated under reduced pressure to give a syrup.

The syrup was allowed to stand at room temperature, resulting in the formation of crystals. A suitable crystal was selected for single-crystal X-ray diffraction analysis.

## Refinement

Crystal data, data collection and structure refinement details are summarized in Table 2 ▸.

## Supplementary Material

Crystal structure: contains datablock(s) I. DOI: 10.1107/S2414314626011247/vm4088sup1.cif

Structure factors: contains datablock(s) I. DOI: 10.1107/S2414314626011247/vm4088Isup2.hkl

CCDC reference: 2597473

Additional supporting information:  crystallographic information; 3D view; checkCIF report

## Figures and Tables

**Table 1:** Hydrogen-bond geometry (Å, °)

*D*—H⋯*A*	*D*—H	H⋯*A*	*D*⋯*A*	*D*—H⋯*A*
O1—H1⋯O1^i^	0.82	1.97	2.776 (2)	166
O7—H7⋯O7^ii^	0.82	1.94	2.738 (2)	164

Symmetry codes: (i) 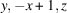; (ii) 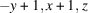.

**Table 2:** Experimental details

Crystal data	
Chemical formula	C_12_H_20_O_6_
*M*_r_	260.28
Crystal system, space group	Tetragonal, *P*4
Temperature (K)	296
*a*, *c* (Å)	21.5539 (14), 5.9556 (2)
*V* (Å^3^)	2766.8 (4)
*Z*	8
Radiation type	Cu *K*α
μ (mm^−1^)	0.84
Crystal size (mm)	0.10 × 0.10 × 0.10

Data collection	
Diffractometer	Rigaku R-AXIS RAPID
Absorption correction	Multi-scan (*ABSCOR*; Rigaku, 1995 ▸)
*T*_min_, *T*_max_	0.713, 1.000
No. of measured, independent and observed [*I* > 2σ(*I*)] reflections	31542, 4815, 4338
*R*_int_	0.042
(sin θ/λ)_max_ (Å^−1^)	0.602

Refinement	
*R*[*F*^2^ > 2σ(*F*^2^)], *wR*(*F*^2^), *S*	0.034, 0.085, 1.05
No. of reflections	4815
No. of parameters	343
No. of restraints	1
H-atom treatment	H atoms treated by a mixture of independent and constrained refinement
Δρ_max_, Δρ_min_ (e Å^−3^)	0.10, −0.14
Absolute structure	Flack *x* determined using 1623 quotients [(*I*^+^)−(*I*^−^)]/[(*I*^+^)+(*I*^−^)] (Parsons *et al.*, 2013 ▸)
Absolute structure parameter	0.08 (6)

Computer programs: *RAPID-AUTO* (Rigaku, 2009 ▸), *SHELXT2018/2* (Sheldrick, 2015*a* ▸), *SHELXL2018/3* (Sheldrick, 2015*b* ▸) and *OLEX2* 1.3 (Dolomanov *et al.*, 2009 ▸).

## full crystallographic data

### 2,3:4,5-Di-O-isopropylidene-β-L-fructopyranose Crystal data

**Table:**

C_12_H_20_O_6_	*D*_x_ = 1.250 Mg m^−^^3^
*M**_r_* = 260.28	Cu *K*α radiation, λ = 1.5418 Å
Tetragonal, *P*4	Cell parameters from 28410 reflections
*a* = 21.5539 (14) Å	θ = 4.1–68.1°
*c* = 5.9556 (2) Å	µ = 0.84 mm^−^^1^
*V* = 2766.8 (4) Å^3^	*T* = 296 K
*Z* = 8	Block, clear light colourless
*F*(000) = 1120	0.1 × 0.1 × 0.1 mm

### 2,3:4,5-Di-O-isopropylidene-β-L-fructopyranose Data collection

**Table:**

Rigaku R-AXIS RAPID diffractometer	4338 reflections with *I* > 2σ(*I*)
ω scans	*R*_int_ = 0.042
Absorption correction: multi-scan (*ABSCOR*; Rigaku, 1995)	θ_max_ = 68.1°, θ_min_ = 4.1°
*T*_min_ = 0.713, *T*_max_ = 1.000	*h* = −25→25
31542 measured reflections	*k* = −25→25
4815 independent reflections	*l* = −7→6

### 2,3:4,5-Di-O-isopropylidene-β-L-fructopyranose Refinement

**Table:**

Refinement on *F*^2^	Hydrogen site location: mixed
Least-squares matrix: full	H atoms treated by a mixture of independent and constrained refinement
*R*[*F*^2^ > 2σ(*F*^2^)] = 0.034	*w* = 1/[σ^2^(*F*_o_^2^) + (0.0473*P*)^2^ + 0.1529*P*] where *P* = (*F*_o_^2^ + 2*F*_c_^2^)/3
*wR*(*F*^2^) = 0.085	(Δ/σ)_max_ < 0.001
*S* = 1.05	Δρ_max_ = 0.10 e Å^−^^3^
4815 reflections	Δρ_min_ = −0.14 e Å^−^^3^
343 parameters	Absolute structure: Flack *x* determined using 1623 quotients [(*I*^+^)-(*I*^-^)]/[(*I*^+^)+(*I*^-^)] (Parsons *et al.*, 2013)
1 restraint	Absolute structure parameter: 0.08 (6)
Primary atom site location: dual	

### 2,3:4,5-Di-O-isopropylidene-β-L-fructopyranose Special details

**Table:**

Geometry. All esds (except the esd in the dihedral angle between two l.s. planes) are estimated using the full covariance matrix. The cell esds are taken into account individually in the estimation of esds in distances, angles and torsion angles; correlations between esds in cell parameters are only used when they are defined by crystal symmetry. An approximate (isotropic) treatment of cell esds is used for estimating esds involving l.s. planes.
Refinement. The H atoms attached to C1 were located in a difference-Fourier map and refined freely with isotropic displacement parameters. The remaining C-bound H atoms were placed in geometrically calculated positions and refined using a riding model, with C—H distances of 0.96–0.98 Å. Hydroxy H atoms were placed at calculated positions with O—H distances of 0.82 Å and refined as rotating groups.The isotropic displacement parameters were set at *U*_iso_(H) = 1.2*U*_eq_(C) for methine and methylene H atoms and 1.5*U*_eq_(C, O) for methyl and hydroxy H atoms. The absolute structure was determined using 1623 quotients of the form [(I+) - (I–)]/[(I+) + (I–)], giving a Flack parameter of 0.08 (6).

### 2,3:4,5-Di-O-isopropylidene-β-L-fructopyranose Fractional atomic coordinates and isotropic or equivalent isotropic displacement parameters (Å^2^)

**Table:**

	*x*	*y*	*z*	*U*_iso_*/*U*_eq_	
O1	0.40902 (7)	0.50423 (7)	0.0959 (4)	0.0585 (5)	
H1	0.432457	0.534186	0.098235	0.088*	
O2	0.35138 (7)	0.61784 (7)	0.1992 (3)	0.0504 (4)	
O3	0.27266 (7)	0.61725 (7)	−0.0510 (3)	0.0549 (4)	
O4	0.21488 (8)	0.47003 (8)	0.1486 (4)	0.0624 (5)	
O5	0.15362 (10)	0.51805 (10)	0.4038 (5)	0.0920 (8)	
O6	0.28592 (7)	0.56325 (8)	0.4383 (3)	0.0559 (4)	
C1	0.36612 (12)	0.50913 (12)	0.2726 (5)	0.0549 (6)	
H1A	0.3841 (13)	0.5149 (13)	0.424 (6)	0.075 (9)*	
H1B	0.3434 (12)	0.4712 (12)	0.287 (5)	0.058 (7)*	
C2	0.31924 (10)	0.56081 (10)	0.2366 (5)	0.0470 (5)	
C3	0.27778 (11)	0.55522 (10)	0.0291 (4)	0.0484 (6)	
H3	0.297236	0.528595	−0.084240	0.058*	
C4	0.21237 (11)	0.53327 (10)	0.0800 (5)	0.0535 (6)	
H4	0.185960	0.537717	−0.052962	0.064*	
C5	0.18308 (11)	0.56620 (11)	0.2810 (5)	0.0597 (7)	
H5	0.152024	0.596113	0.228776	0.072*	
C6	0.23011 (11)	0.59846 (12)	0.4263 (5)	0.0582 (7)	
H6A	0.238985	0.639176	0.364892	0.070*	
H6B	0.213313	0.603936	0.576146	0.070*	
C7	0.33040 (11)	0.64682 (11)	−0.0034 (5)	0.0562 (7)	
C8	0.37644 (13)	0.63633 (15)	−0.1909 (5)	0.0719 (8)	
H8A	0.415912	0.653440	−0.149266	0.108*	
H8B	0.361862	0.656253	−0.325109	0.108*	
H8C	0.380786	0.592616	−0.217617	0.108*	
C9	0.31850 (13)	0.71473 (12)	0.0394 (7)	0.0834 (11)	
H9A	0.289930	0.719124	0.162000	0.125*	
H9B	0.301144	0.733340	−0.092940	0.125*	
H9C	0.356844	0.734969	0.076231	0.125*	
C10	0.16479 (14)	0.46018 (13)	0.2986 (7)	0.0756 (9)	
C11	0.10731 (17)	0.44069 (19)	0.1687 (9)	0.1144 (15)	
H11A	0.097753	0.471569	0.057846	0.172*	
H11B	0.072988	0.436517	0.270360	0.172*	
H11C	0.114875	0.401671	0.095992	0.172*	
C12	0.1840 (2)	0.4143 (2)	0.4743 (9)	0.1260 (17)	
H12A	0.191654	0.374784	0.405220	0.189*	
H12B	0.151546	0.410152	0.583644	0.189*	
H12C	0.221174	0.428538	0.546742	0.189*	
O7	0.03136 (7)	0.91583 (7)	−0.1048 (4)	0.0567 (5)	
H7	0.053714	0.946614	−0.102355	0.085*	
O8	0.15807 (7)	0.89231 (7)	−0.1922 (3)	0.0479 (4)	
O9	0.17575 (7)	0.81666 (6)	0.0631 (3)	0.0487 (4)	
O10	0.05030 (7)	0.72325 (7)	−0.1521 (3)	0.0553 (5)	
O11	0.11500 (8)	0.67555 (9)	−0.3981 (4)	0.0794 (7)	
O12	0.12620 (8)	0.81417 (8)	−0.4345 (3)	0.0532 (4)	
C13	0.04996 (10)	0.87641 (11)	−0.2817 (5)	0.0535 (6)	
H13A	0.052680	0.900266	−0.419495	0.064*	
H13B	0.018856	0.844425	−0.303102	0.064*	
C14	0.11241 (10)	0.84602 (10)	−0.2350 (4)	0.0440 (5)	
C15	0.11551 (10)	0.80518 (10)	−0.0257 (4)	0.0426 (5)	
H15	0.083521	0.817439	0.082449	0.051*	
C16	0.11134 (10)	0.73627 (10)	−0.0755 (5)	0.0469 (5)	
H16	0.120573	0.712117	0.059899	0.056*	
C17	0.15305 (10)	0.71584 (10)	−0.2687 (5)	0.0509 (6)	
H17	0.188941	0.693064	−0.210220	0.061*	
C18	0.17449 (11)	0.76945 (11)	−0.4106 (5)	0.0544 (6)	
H18A	0.210346	0.788742	−0.341223	0.065*	
H18B	0.186747	0.754435	−0.557608	0.065*	
C19	0.18940 (11)	0.88016 (10)	0.0158 (5)	0.0505 (6)	
C20	0.16511 (14)	0.92255 (11)	0.1983 (5)	0.0657 (7)	
H20A	0.121652	0.914952	0.220428	0.099*	
H20B	0.171210	0.964985	0.154611	0.099*	
H20C	0.187057	0.914596	0.335560	0.099*	
C21	0.25853 (12)	0.88619 (13)	−0.0166 (7)	0.0761 (9)	
H21A	0.279550	0.871490	0.115211	0.114*	
H21B	0.268931	0.928947	−0.041539	0.114*	
H21C	0.271117	0.861982	−0.143967	0.114*	
C22	0.05386 (11)	0.67444 (12)	−0.3135 (5)	0.0594 (7)	
C23	0.01034 (16)	0.68932 (19)	−0.5024 (7)	0.0958 (12)	
H23A	−0.031670	0.687308	−0.449319	0.144*	
H23B	0.016001	0.659852	−0.621512	0.144*	
H23C	0.018817	0.730331	−0.557299	0.144*	
C24	0.0415 (2)	0.61259 (14)	−0.2036 (8)	0.1050 (13)	
H24A	0.070346	0.606422	−0.082786	0.157*	
H24B	0.046385	0.580063	−0.312281	0.157*	
H24C	−0.000060	0.611932	−0.145751	0.157*	

### 2,3:4,5-Di-O-isopropylidene-β-L-fructopyranose Atomic displacement parameters (Å^2^)

**Table:**

	*U*^11^	*U*^22^	*U*^33^	*U*^12^	*U*^13^	*U*^23^
O1	0.0440 (8)	0.0432 (8)	0.0883 (14)	0.0012 (7)	0.0050 (9)	−0.0127 (9)
O2	0.0486 (8)	0.0469 (8)	0.0558 (11)	0.0018 (6)	−0.0156 (8)	−0.0080 (8)
O3	0.0485 (9)	0.0519 (9)	0.0641 (12)	−0.0020 (7)	−0.0187 (8)	−0.0010 (9)
O4	0.0646 (11)	0.0494 (9)	0.0733 (13)	−0.0023 (8)	0.0091 (9)	−0.0113 (9)
O5	0.0887 (14)	0.0776 (13)	0.110 (2)	−0.0151 (11)	0.0468 (14)	−0.0262 (14)
O6	0.0527 (9)	0.0655 (10)	0.0494 (11)	0.0125 (8)	−0.0041 (8)	−0.0095 (9)
C1	0.0501 (13)	0.0508 (14)	0.0639 (19)	0.0094 (11)	−0.0042 (13)	−0.0030 (13)
C2	0.0460 (12)	0.0439 (11)	0.0511 (15)	0.0050 (9)	−0.0039 (11)	−0.0099 (11)
C3	0.0480 (12)	0.0445 (12)	0.0526 (16)	0.0037 (9)	−0.0060 (11)	−0.0132 (11)
C4	0.0475 (12)	0.0510 (13)	0.0621 (16)	−0.0005 (10)	−0.0094 (12)	−0.0105 (12)
C5	0.0466 (12)	0.0561 (14)	0.076 (2)	0.0067 (10)	0.0006 (13)	−0.0143 (14)
C6	0.0528 (13)	0.0603 (14)	0.0615 (17)	0.0138 (11)	−0.0010 (13)	−0.0193 (13)
C7	0.0494 (13)	0.0516 (13)	0.0676 (19)	−0.0022 (10)	−0.0202 (13)	−0.0004 (12)
C8	0.0613 (16)	0.087 (2)	0.067 (2)	−0.0149 (14)	−0.0109 (15)	0.0084 (16)
C9	0.0724 (18)	0.0483 (14)	0.130 (3)	−0.0013 (12)	−0.036 (2)	−0.0008 (18)
C10	0.0751 (18)	0.0645 (17)	0.087 (2)	−0.0077 (14)	0.0223 (17)	−0.0086 (17)
C11	0.089 (2)	0.106 (3)	0.148 (4)	−0.039 (2)	0.014 (3)	−0.022 (3)
C12	0.153 (4)	0.117 (3)	0.108 (4)	0.021 (3)	0.042 (3)	0.034 (3)
O7	0.0417 (8)	0.0393 (8)	0.0891 (14)	0.0014 (6)	0.0105 (9)	−0.0046 (9)
O8	0.0481 (8)	0.0449 (8)	0.0509 (11)	0.0005 (6)	0.0003 (7)	0.0101 (7)
O9	0.0538 (9)	0.0412 (8)	0.0509 (10)	−0.0006 (6)	−0.0087 (8)	0.0055 (8)
O10	0.0466 (8)	0.0530 (9)	0.0664 (13)	−0.0019 (7)	0.0024 (8)	−0.0114 (9)
O11	0.0554 (10)	0.0810 (13)	0.1018 (18)	−0.0014 (9)	0.0055 (11)	−0.0474 (13)
O12	0.0612 (10)	0.0569 (9)	0.0416 (10)	0.0164 (8)	0.0011 (8)	0.0001 (8)
C13	0.0476 (12)	0.0489 (12)	0.0641 (18)	0.0096 (10)	−0.0036 (12)	0.0030 (12)
C14	0.0445 (11)	0.0418 (11)	0.0457 (14)	0.0055 (9)	0.0017 (10)	0.0010 (10)
C15	0.0438 (11)	0.0407 (11)	0.0434 (14)	0.0030 (9)	0.0033 (10)	−0.0017 (10)
C16	0.0495 (12)	0.0396 (11)	0.0517 (15)	0.0019 (9)	−0.0038 (11)	0.0034 (11)
C17	0.0457 (11)	0.0448 (11)	0.0622 (16)	0.0091 (9)	−0.0038 (12)	−0.0085 (12)
C18	0.0531 (13)	0.0604 (14)	0.0498 (16)	0.0163 (11)	0.0059 (12)	−0.0064 (13)
C19	0.0543 (13)	0.0429 (11)	0.0544 (16)	−0.0024 (10)	−0.0054 (11)	0.0091 (11)
C20	0.0903 (19)	0.0469 (13)	0.0600 (19)	−0.0064 (13)	−0.0077 (16)	−0.0030 (13)
C21	0.0558 (15)	0.0677 (17)	0.105 (3)	−0.0085 (13)	−0.0138 (16)	0.0162 (17)
C22	0.0537 (13)	0.0571 (14)	0.0675 (18)	−0.0004 (11)	−0.0026 (13)	−0.0128 (13)
C23	0.080 (2)	0.127 (3)	0.081 (3)	0.009 (2)	−0.0206 (19)	−0.024 (2)
C24	0.141 (3)	0.0592 (18)	0.115 (3)	−0.0181 (19)	0.004 (3)	−0.011 (2)

### 2,3:4,5-Di-O-isopropylidene-β-L-fructopyranose Geometric parameters (Å, º)

**Table:**

O1—H1	0.8200	O7—H7	0.8200
O1—C1	1.405 (3)	O7—C13	1.412 (3)
O2—C2	1.429 (3)	O8—C14	1.425 (3)
O2—C7	1.432 (3)	O8—C19	1.435 (3)
O3—C3	1.424 (3)	O9—C15	1.424 (3)
O3—C7	1.427 (3)	O9—C19	1.428 (3)
O4—C4	1.424 (3)	O10—C16	1.421 (3)
O4—C10	1.417 (4)	O10—C22	1.427 (3)
O5—C5	1.419 (3)	O11—C17	1.422 (3)
O5—C10	1.417 (3)	O11—C22	1.411 (3)
O6—C2	1.400 (3)	O12—C14	1.404 (3)
O6—C6	1.424 (3)	O12—C18	1.426 (3)
C1—H1A	0.99 (4)	C13—H13A	0.9700
C1—H1B	0.96 (3)	C13—H13B	0.9700
C1—C2	1.519 (3)	C13—C14	1.522 (3)
C2—C3	1.530 (3)	C14—C15	1.527 (3)
C3—H3	0.9800	C15—H15	0.9800
C3—C4	1.518 (3)	C15—C16	1.517 (3)
C4—H4	0.9800	C16—H16	0.9800
C4—C5	1.528 (4)	C16—C17	1.525 (4)
C5—H5	0.9800	C17—H17	0.9800
C5—C6	1.503 (4)	C17—C18	1.505 (4)
C6—H6A	0.9700	C18—H18A	0.9700
C6—H6B	0.9700	C18—H18B	0.9700
C7—C8	1.511 (4)	C19—C20	1.513 (4)
C7—C9	1.508 (4)	C19—C21	1.508 (3)
C8—H8A	0.9600	C20—H20A	0.9600
C8—H8B	0.9600	C20—H20B	0.9600
C8—H8C	0.9600	C20—H20C	0.9600
C9—H9A	0.9600	C21—H21A	0.9600
C9—H9B	0.9600	C21—H21B	0.9600
C9—H9C	0.9600	C21—H21C	0.9600
C10—C11	1.520 (5)	C22—C23	1.499 (4)
C10—C12	1.499 (5)	C22—C24	1.509 (4)
C11—H11A	0.9600	C23—H23A	0.9600
C11—H11B	0.9600	C23—H23B	0.9600
C11—H11C	0.9600	C23—H23C	0.9600
C12—H12A	0.9600	C24—H24A	0.9600
C12—H12B	0.9600	C24—H24B	0.9600
C12—H12C	0.9600	C24—H24C	0.9600
			
C1—O1—H1	109.5	C13—O7—H7	109.5
C2—O2—C7	110.72 (17)	C14—O8—C19	110.60 (17)
C3—O3—C7	106.58 (16)	C15—O9—C19	106.32 (16)
C10—O4—C4	107.17 (19)	C16—O10—C22	108.20 (17)
C10—O5—C5	109.9 (2)	C22—O11—C17	110.8 (2)
C2—O6—C6	114.2 (2)	C14—O12—C18	113.63 (18)
O1—C1—H1A	115.6 (17)	O7—C13—H13A	109.2
O1—C1—H1B	109.7 (17)	O7—C13—H13B	109.2
O1—C1—C2	112.8 (2)	O7—C13—C14	111.9 (2)
H1A—C1—H1B	103 (2)	H13A—C13—H13B	107.9
C2—C1—H1A	107.3 (17)	C14—C13—H13A	109.2
C2—C1—H1B	107.4 (15)	C14—C13—H13B	109.2
O2—C2—C1	109.29 (19)	O8—C14—C13	110.01 (17)
O2—C2—C3	103.0 (2)	O8—C14—C15	103.13 (18)
O6—C2—O2	110.52 (18)	O12—C14—O8	110.35 (18)
O6—C2—C1	104.3 (2)	O12—C14—C13	104.1 (2)
O6—C2—C3	113.35 (17)	O12—C14—C15	113.55 (16)
C1—C2—C3	116.4 (2)	C13—C14—C15	115.8 (2)
O3—C3—C2	103.99 (17)	O9—C15—C14	104.07 (17)
O3—C3—H3	110.6	O9—C15—H15	110.5
O3—C3—C4	106.71 (18)	O9—C15—C16	107.26 (17)
C2—C3—H3	110.6	C14—C15—H15	110.5
C4—C3—C2	113.9 (2)	C16—C15—C14	113.7 (2)
C4—C3—H3	110.6	C16—C15—H15	110.5
O4—C4—C3	108.69 (18)	O10—C16—C15	108.11 (17)
O4—C4—H4	110.3	O10—C16—H16	110.3
O4—C4—C5	103.6 (2)	O10—C16—C17	104.3 (2)
C3—C4—H4	110.3	C15—C16—H16	110.3
C3—C4—C5	113.30 (19)	C15—C16—C17	113.26 (19)
C5—C4—H4	110.3	C17—C16—H16	110.3
O5—C5—C4	104.4 (2)	O11—C17—C16	104.21 (19)
O5—C5—H5	109.8	O11—C17—H17	110.0
O5—C5—C6	110.1 (3)	O11—C17—C18	110.0 (2)
C4—C5—H5	109.8	C16—C17—H17	110.0
C6—C5—C4	112.8 (2)	C18—C17—C16	112.53 (18)
C6—C5—H5	109.8	C18—C17—H17	110.0
O6—C6—C5	110.6 (2)	O12—C18—C17	110.6 (2)
O6—C6—H6A	109.5	O12—C18—H18A	109.5
O6—C6—H6B	109.5	O12—C18—H18B	109.5
C5—C6—H6A	109.5	C17—C18—H18A	109.5
C5—C6—H6B	109.5	C17—C18—H18B	109.5
H6A—C6—H6B	108.1	H18A—C18—H18B	108.1
O2—C7—C8	110.5 (2)	O8—C19—C20	110.30 (19)
O2—C7—C9	109.6 (2)	O8—C19—C21	109.8 (2)
O3—C7—O2	104.4 (2)	O9—C19—O8	104.38 (18)
O3—C7—C8	111.0 (2)	O9—C19—C20	111.4 (2)
O3—C7—C9	108.60 (19)	O9—C19—C21	108.15 (19)
C9—C7—C8	112.4 (3)	C21—C19—C20	112.4 (2)
C7—C8—H8A	109.5	C19—C20—H20A	109.5
C7—C8—H8B	109.5	C19—C20—H20B	109.5
C7—C8—H8C	109.5	C19—C20—H20C	109.5
H8A—C8—H8B	109.5	H20A—C20—H20B	109.5
H8A—C8—H8C	109.5	H20A—C20—H20C	109.5
H8B—C8—H8C	109.5	H20B—C20—H20C	109.5
C7—C9—H9A	109.5	C19—C21—H21A	109.5
C7—C9—H9B	109.5	C19—C21—H21B	109.5
C7—C9—H9C	109.5	C19—C21—H21C	109.5
H9A—C9—H9B	109.5	H21A—C21—H21B	109.5
H9A—C9—H9C	109.5	H21A—C21—H21C	109.5
H9B—C9—H9C	109.5	H21B—C21—H21C	109.5
O4—C10—C11	110.0 (3)	O10—C22—C23	108.3 (2)
O4—C10—C12	109.2 (3)	O10—C22—C24	110.5 (3)
O5—C10—O4	106.0 (2)	O11—C22—O10	106.16 (18)
O5—C10—C11	109.3 (3)	O11—C22—C23	108.2 (3)
O5—C10—C12	108.6 (3)	O11—C22—C24	109.5 (3)
C12—C10—C11	113.5 (3)	C23—C22—C24	113.8 (3)
C10—C11—H11A	109.5	C22—C23—H23A	109.5
C10—C11—H11B	109.5	C22—C23—H23B	109.5
C10—C11—H11C	109.5	C22—C23—H23C	109.5
H11A—C11—H11B	109.5	H23A—C23—H23B	109.5
H11A—C11—H11C	109.5	H23A—C23—H23C	109.5
H11B—C11—H11C	109.5	H23B—C23—H23C	109.5
C10—C12—H12A	109.5	C22—C24—H24A	109.5
C10—C12—H12B	109.5	C22—C24—H24B	109.5
C10—C12—H12C	109.5	C22—C24—H24C	109.5
H12A—C12—H12B	109.5	H24A—C24—H24B	109.5
H12A—C12—H12C	109.5	H24A—C24—H24C	109.5
H12B—C12—H12C	109.5	H24B—C24—H24C	109.5
			
O1—C1—C2—O2	−55.0 (3)	O7—C13—C14—O8	−56.0 (3)
O1—C1—C2—O6	−173.2 (2)	O7—C13—C14—O12	−174.25 (19)
O1—C1—C2—C3	61.1 (3)	O7—C13—C14—C15	60.4 (3)
O2—C2—C3—O3	−21.9 (2)	O8—C14—C15—O9	−22.4 (2)
O2—C2—C3—C4	−137.70 (19)	O8—C14—C15—C16	−138.74 (18)
O3—C3—C4—O4	176.7 (2)	O9—C15—C16—O10	176.66 (19)
O3—C3—C4—C5	−68.7 (3)	O9—C15—C16—C17	−68.3 (3)
O4—C4—C5—O5	−19.1 (3)	O10—C16—C17—O11	−18.7 (2)
O4—C4—C5—C6	100.3 (2)	O10—C16—C17—C18	100.4 (2)
O5—C5—C6—O6	79.6 (3)	O11—C17—C18—O12	78.1 (2)
O6—C2—C3—O3	97.5 (2)	O12—C14—C15—O9	97.1 (2)
O6—C2—C3—C4	−18.3 (3)	O12—C14—C15—C16	−19.3 (3)
C1—C2—C3—O3	−141.5 (2)	C13—C14—C15—O9	−142.6 (2)
C1—C2—C3—C4	102.8 (2)	C13—C14—C15—C16	101.0 (2)
C2—O2—C7—O3	17.5 (2)	C14—O8—C19—O9	17.3 (2)
C2—O2—C7—C8	−101.9 (2)	C14—O8—C19—C20	−102.5 (2)
C2—O2—C7—C9	133.6 (2)	C14—O8—C19—C21	133.0 (2)
C2—O6—C6—C5	68.7 (3)	C14—O12—C18—C17	69.1 (3)
C2—C3—C4—O4	−69.1 (3)	C14—C15—C16—O10	−68.8 (2)
C2—C3—C4—C5	45.5 (3)	C14—C15—C16—C17	46.2 (3)
C3—O3—C7—O2	−32.0 (2)	C15—O9—C19—O8	−32.0 (2)
C3—O3—C7—C8	87.0 (3)	C15—O9—C19—C20	87.0 (2)
C3—O3—C7—C9	−148.8 (3)	C15—O9—C19—C21	−148.9 (2)
C3—C4—C5—O5	−136.8 (2)	C15—C16—C17—O11	−136.0 (2)
C3—C4—C5—C6	−17.3 (3)	C15—C16—C17—C18	−16.9 (3)
C4—O4—C10—O5	−28.9 (3)	C16—O10—C22—O11	−22.9 (3)
C4—O4—C10—C11	89.2 (3)	C16—O10—C22—C23	−138.9 (2)
C4—O4—C10—C12	−145.7 (3)	C16—O10—C22—C24	95.8 (3)
C4—C5—C6—O6	−36.5 (3)	C16—C17—C18—O12	−37.6 (3)
C5—O5—C10—O4	16.0 (3)	C17—O11—C22—O10	10.2 (3)
C5—O5—C10—C11	−102.5 (3)	C17—O11—C22—C23	126.3 (3)
C5—O5—C10—C12	133.2 (3)	C17—O11—C22—C24	−109.0 (3)
C6—O6—C2—O2	76.4 (2)	C18—O12—C14—O8	77.3 (2)
C6—O6—C2—C1	−166.2 (2)	C18—O12—C14—C13	−164.68 (19)
C6—O6—C2—C3	−38.6 (3)	C18—O12—C14—C15	−37.9 (3)
C7—O2—C2—O6	−118.7 (2)	C19—O8—C14—O12	−118.53 (19)
C7—O2—C2—C1	127.1 (2)	C19—O8—C14—C13	127.2 (2)
C7—O2—C2—C3	2.7 (2)	C19—O8—C14—C15	3.1 (2)
C7—O3—C3—C2	33.7 (2)	C19—O9—C15—C14	33.9 (2)
C7—O3—C3—C4	154.4 (2)	C19—O9—C15—C16	154.7 (2)
C10—O4—C4—C3	150.2 (2)	C22—O10—C16—C15	146.5 (2)
C10—O4—C4—C5	29.5 (3)	C22—O10—C16—C17	25.7 (2)
C10—O5—C5—C4	2.1 (3)	C22—O11—C17—C16	5.3 (3)
C10—O5—C5—C6	−119.2 (3)	C22—O11—C17—C18	−115.6 (2)

### 2,3:4,5-Di-O-isopropylidene-β-L-fructopyranose Hydrogen-bond geometry (Å, º)

**Table:**

*D*—H···*A*	*D*—H	H···*A*	*D*···*A*	*D*—H···*A*
O1—H1···O1^i^	0.82	1.97	2.776 (2)	166
O7—H7···O7^ii^	0.82	1.94	2.738 (2)	164

Symmetry codes: (i) *y*, −*x*+1, *z*; (ii) −*y*+1, *x*+1, *z*.
